# Prognosis and Risk Factors for Deterioration in Patients Admitted to a Medical Emergency Department

**DOI:** 10.1371/journal.pone.0094649

**Published:** 2014-04-09

**Authors:** Daniel Pilsgaard Henriksen, Mikkel Brabrand, Annmarie Touborg Lassen

**Affiliations:** 1 Department of Emergency Medicine, Odense University Hospital, Odense, Denmark; 2 Department of Medicine, Sydvestjysk Sygehus, Esbjerg, Denmark; Health Protection Agency, United Kingdom

## Abstract

**Objective:**

Patients that initially appear stable on arrival to the hospital often have less intensive monitoring of their vital signs, possibly leading to excess mortality. The aim was to describe risk factors for deterioration in vital signs and the related prognosis among patients with normal vital signs at arrival to a medical emergency department (MED).

**Design and setting:**

Single-centre, retrospective cohort study of all patients admitted to the MED from September 2010-August 2011.

**Subjects:**

Patients were included when their vital signs (systolic blood pressure, pulse rate, respiratory rate, Glasgow Coma Scale, oxygen saturation and temperature) were within the normal range at arrival. Deterioration was defined as a deviation from the defined normal range 2–24 hours after arrival.

**Results:**

4292 of the 6257 (68.6%) admitted to the MED had a full set of vital signs at first presentation, 1440/4292 (33.6%) had all normal vital signs and were included in study, 44.0% were male, median age 64 years (5th/95th percentile: 21–90 years) and 446/1440 (31.0%) deteriorated within 24 hours. Independent risk factors for deterioration included age 65–84 years odds ratio (OR): 1.79 (95% confidence interval [CI]: 1.27–2.52), 85+ years OR 1.67 (95% CI: 1.10–2.55), Do-not-attempt-to-resuscitate order OR 3.76 (95% CI: 1.37–10.31) and admission from the open general ED OR 1.35 (95% CI: 1.07–1.71). Thirty-day mortality was 7.9% (95% CI: 5.5–10.7%) among deteriorating patients and 1.9% (95% CI: 1.2–3.0%) among the non-deteriorating, hazard ratio 4.11 (95% CI: 2.38–7.10).

**Conclusions:**

Among acutely admitted medical patients who arrive with normal vital signs, 31.0% showed signs of deterioration within 24 hours. Risk factors included old age, Do-not-attempt-to-resuscitate order, admission from the open general ED. Thirty-day mortality among patients with deterioration was four times higher than among non-deteriorating patients. Further research is needed to determine whether intensified monitoring of vital signs can help to prevent deterioration or mortality among medical emergency patients.

## Introduction

Most emergency departments (ED) are at constant risk of overcrowding [1]. As a solution to this, specialised admission units for acute medical patients have been implemented, which in turn reduced waiting time, length of stay, and mortality [2]. Patients who appear stable at arrival often receive low priority from the nursing staff and their vital signs are insufficiently recorded[3]–[6]. However, clinical deterioration has been shown to be an independent predictor of mortality and early identification could improve prognosis [1], [7], [8].

There is surprisingly little knowledge of how to monitor and deal with deteriorating patients[3]–[7], [9], [10]. However, increased knowledge could provide information to “post triage” follow-up procedures in the emergency departments and admission units and possibly lead to reduced morbidity and mortality.

The aim of our study was to describe the risk factors and prognosis of patients that arrive with normal vital signs but clinically deteriorate within the first 24 hours after arrival.

## Materials and Methods

### Study Design and Setting

We conducted a retrospective cohort study of all consecutive adult (≥15 years) patients admitted to the medical ED at Odense University Hospital over a one-year period (1 September 2010 through 31 August 2011). The medical ED serves a population of 235,000 adults and as a medical admission unit for the following medical specialities: general internal medicine, infectious diseases, gastroenterology, geriatric medicine, rheumatology, endocrinology and respiratory medicine. The medical ED receives all acutely admitted medical patients from either primary care or from the hospitals open mixed medical-surgical ED.

Initial vital signs were routinely collected upon arrival to the hospital (blood pressure, heart rate, respiratory rate, rectal temperature, peripheral oxygen saturation, recorded oxygen therapy, and Glasgow Coma Scale). Any later vital signs were collected as deemed necessary by the attending physician, but all vital signs were routinely recorded at least every eight hours. There were no interventions related to the study, and all patients received care following the standard operating procedures. We excluded patients unidentified throughout the entire course of admission, patients without a Danish civil registration number, and patients without any recorded vital signs or with initial vital signs recorded >2 hours after arrival to the hospital. We included only the first admission of each patient, within the study period.

### Variables/Definitions

We defined normal vital signs as systolic blood pressure >100 mmHg, heart rate ≥50/min and ≤90/min, respiratory rate ≥8/min and ≤20/min, rectal temperature ≥36.0° and ≤38.0°Celsius, oxygen saturation ≥94% (≥90% in patients with a history of chronic obstructive pulmonary disease [COPD]) and no need for oxygen therapy at arrival to the hospital and a Glasgow Scale Score of 15 or registered as awake and alert. Clinical deterioration was defined as vital signs beyond the definition of normal.

Immunosuppression was defined as one or more of the following identified before the current admission:

1) A moderate to high intake of immunosuppressant medication (corticosteroids or antineoplastic and immunomodulating agents) 2) Primary immunodeficiency diseases; 3) Acquired Immune Deficiency Syndrome defining diseases; 4) A newly diagnosed malignancy registered in the Danish Cancer Registry, up to a year prior to admission; 5) Diagnosis of organ transplantation.

Alcoholism-related condition was defined as at least one of the following before the current admission:

1) A redeemed prescription of Disulfiram from 2007 and onwards; 2) ≥2 admissions with a discharge diagnosis of an acute alcohol episode; 3) At least one admission with a discharge diagnosis of a chronic alcohol related diagnosis; 4) A registration in The Danish National Register of Alcohol Abuse Treatment.

Comorbidity was reported by Charlson Comorbidity Index based on discharge diagnoses in the last 10 years and up to seven days prior the current admission.

Patients were coded as Do-Not-Attempt-Resuscitation (DNAR) if they had a DNAR order placed in the medical record within 24 hours of presentation. If a patient was characterised as having a terminal illness or moribund, we defined them as having a DNAR order as well.

### Data Sources/Measurement

Vital signs registered upon arrival to the department and in the following 24 hours were extracted electronically from the patient records. For each patient, we extracted data from the Danish National Patient Register [11] (comorbidity and discharge diagnoses), the Danish Civil Persons Register [12] (vital status [dead or alive] and emigration status), Odense University Pharmacoepidemiological Database [13], the Danish National Cancer Register [14] and the Danish National Alcohol- and Drug Treatment Register, the last three to identify immunosuppression and prior alcoholism-related conditions. All patients were followed up until death, emigration from the country or 30 days after admission, whichever came first.

### Ethics Committee Approval

In compliance with Danish law, the study was notified to and approved by the Danish Data Protection Agency (J No 2008-58-0035), and the access to patient clinical records was approved by the Danish National Board of Health (J No 3-3013-35). No further ethical approval, or consent from participants, is needed for register-based studies in Denmark. Data were anonymised and de-identified prior data analysis.

The reporting of this study conforms to the STROBE statement [15].

### Statistical Methods

Data were presented as medians with 5th and 95th percentiles or proportions with 95% confidence intervals (CI), assuming a binomial distribution. Univariate analysis of baseline characteristics was performed using Chi-squared tests or Mann–Whitney U-tests. A multivariable logistic regression analysis was computed using deterioration as the dependent variable and several predefined potential risk factors for developing clinical deterioration within the first 24 hours after arrival to the hospital, as independent variables. We included each independent variable in the model without assessing their individual association with future clinical deterioration. In each patient we included the first recording of a deterioration among the individual vital signs (systolic blood pressure, pulse rate, GCS, respiratory rate, oxygen saturation and temperature). We computed Kaplan-Meier survival plots of the individual vital signs, where the event was the time of clinical deterioration. Thirty-day mortality was presented as proportions as well as a Kaplan-Meier failure plot and log rank test in a univariate analysis. If a vital sign was missing in the reassessment of the patient, it was defined as if it was within normal range. Statistical analyses were performed with Stata version 13.0 (Stata Corporation LP, Texas, USA).

## Results

### Participants

**Figure 1:**
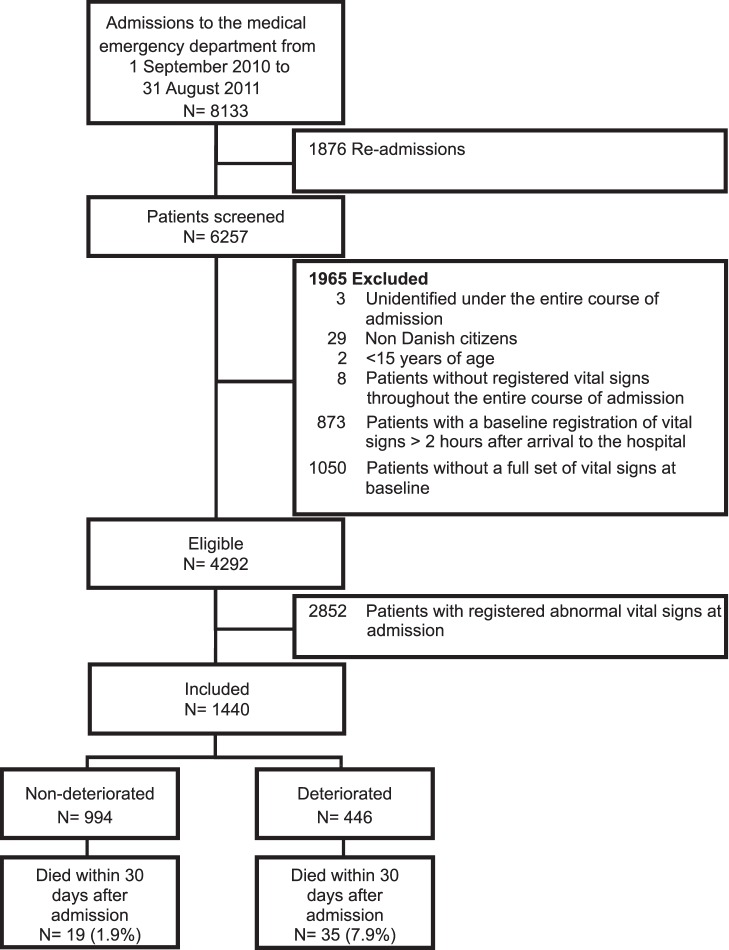
Flow chart of recruitment of patients admitted to the medical emergency department.

A total of 6257 patients were admitted to the medical ED within the study period.,4292/6257 (68.6%) had a full set of vital signs at first presentation,1440/4292 (33.6%) had all normal vital signs and were included (Figure 1). Median age among the included patients was 64 years (21–90 years) and 44.0% were male. The distribution of included patients with comorbidity in the Charlson Comorbidity Index categories 0, 1–2 and >2 were 706 (49.0%), 311 (21.6%), 423 (29.4%), respectively. 446/1440 (31.0%) patients had deteriorating vital signs within the first 24 hours after arrival to the hospital.

### Risk Factors for Deterioration

Independent risk factors for clinical deterioration within the first 24 hours after arrival to the hospital included patients in the age category 65–84 years (odds ratio [OR] 1.79 [95% CI: 1.27–2.52]); patients in the age category 85+ years (OR 1.67 [95% CI: 1.10–2.55]); patients admitted from the open general ED (OR 1.35 [95% CI: 1.07–1.71]) and patients with a DNAR recorded within the first 24 hours after arrival to the hospital (OR 3.76 [95% CI: 1.37–10.31]) (Table 1).

**Table 1:** Risk factors for clinical deterioration within 24 hours in patients admitted to the medical emergency department with normal vital signs registered at arrival.

		Non-deteriorating,N = 994 (69.0%)	DeterioratingN = 446 (31.0%)	Adjusted logisticregression^1^
Gender (%)	Female	551 (68.4)	255 (31.6)	Ref
	Male	443 (69.9)	191 (30.1)	0.90 (0.71–1.13)
Age in categories, years (%)	15–39	239 (75.6)	77 (24.4)	Ref
	40–64	320 (74.6)	109 (25.4)	1.00 (0.70–1.41)
	65–84	318 (62.4)	192 (37.6)	1.79 (1.27–2.52)
	85+	117 (63.2)	68 (36.8)	1.67 (1.10–2.55)
Charlson Comorbidity index (%)	0	516 (73.1)	190 (26.9)	Ref
	1–2	211 (67.8)	100 (32.2)	1.10 (0.81–1.49)
	>2	267 (63.1)	156 (36.9)	1.19 (0.88–1.61)
Immunosuppression (%)	No	889 (69.3)	393 (30.7)	Ref
	Yes	105 (66.5)	53 (33.5)	1.01 (0.70–1.47)
Alcoholism-related conditions (%)	No	901 (69.6)	394 (30.4)	Ref
	Yes	93 (64.1)	52 (35.9)	1.44 (0.98–2.11)
Do-not-attempt-resuscitate order (%)	Resuscitate	988 (69.5)	434 (30.5)	Ref
	Do-not attempt resuscitation	6 (33.3)	12 (66.7)	3.76 (1.37–10.31)
Admission at entry (%)	Primary care admittedmedical ED	642 (71.0)	262 (29.0)	Ref
	Open general ED	352 (65.7)	184 (34.3)	1.35 (1.07–1.71)

1 Adjusted for gender, age in categories, Charlson Comorbidity Index, immunosuppression, alcoholism-related conditions, Do-not-attempt-resuscitation order and admission at entry.

### Discharge Diagnosis

Patients with a discharge diagnosis from the respiratory system had the highest risk of deterioration, (98/153, 64.1%, 95% CI: 55.9–71.6%) followed by discharge diagnoses from the cardiac/circulatory system 30 (35.3% [25.2%–46.4%]) (Table 2).

**Table 2:** Distribution of primary discharge diagnosis from the medical emergency department among patients with- and without registered clinical deterioration within 24 hours after admission.

Discharge Categories (ICD10), N = 1440	N, total	Deteriorating N, (%), [95% CI^1^]	
Infections (A00–B99),	99	31 (31.3% [22.4%–41.4%])	
Anemia and blood disease (D50–D89)	41	13 (31.7% [18.1%–48.1%])	
Endocrine and metabolic (E00–E90),	139	41 (29.5% [22.1%–37.8%])	
Mental and behavioral (F00–F99),	39	11 (28.2% [15.0%–44.9%])	
Nervous system (G00–G99),	12	3 (25.0% [5.5%–57.2%])	
Cardiac/Circulatory system (I00–I99)	85	30 (35.3% [25.2%–46.4%])	
Respiratory system (J00–J99),	153	98 (64.1% [55.9%–71.6%])	
Gastrointestinal system (K00–K93)	112	34 (30.4% [22.0%–39.8%])	
Skin and subcutaneous tissue (L00–L99)	19	2 (10.5% [1.3%–33.1%])	
Musculoskeletal and connective tissue (M00–M99)	129	22 (17.1% [11.0%–24.7%])	
Genitourinary system (N00–N99)	68	23 (33.8% [22.8%–46.3%])	
Toxicologic (T15–T98)	158	48 (30.4% [23.3%–38.2%])	
General signs and symptoms and abnormal clinical and lab findings (R00–R94)	257	63 (24.5% [19.4%–30.2%])	
Other	129	27 (20.9% [14.3%–29.0%])	

1 95% CI: 95%.

### Mortality

**Figure 2:**
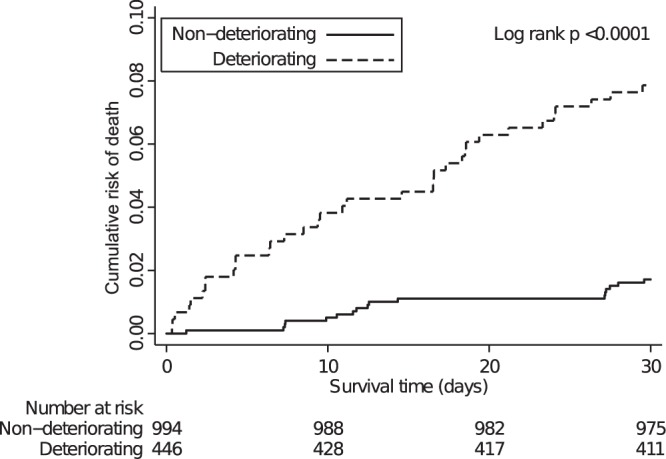
Cumulative risk of death measured in days over a 30-day period among patients with- (dashed line) and without (solid line) recorded clinical deterioration within 24 hours after admission to the medical emergency department.

The overall 30-day mortality of the included patients was 3.8% (95% CI: 2.8–4.9%). In the non-deteriorating group, 19/994 (1.9%, 95% CI: 1.2–3.0%) patients died compared to 35/446 (7.9%, 95% CI: 5.5–10.7%) patients in the deteriorating group (Figure 2), unadjusted hazard ratio 4.11 (95% CI: 2.38–7.10). Of all deteriorating patients, 18/314 (5.7%, 95% CI: 3.4–8.9%) experiencing a single vital sign deteriorating within 24 hours after arrival died within 30 days after admission, 12/99 (12.1%, 95% CI: 6.4–20.2%) with two individual vital sign deteriorating and 5/33 (15.2%, 95% CI: 5.1–31.9%) with three or more individual vital sign deteriorating.

### Vital Signs

**Figure 3:**
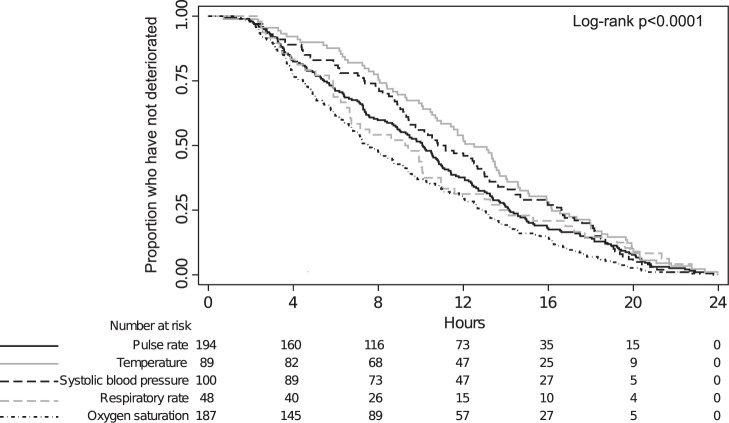
Kaplan Meier plot illustrating the different vital signs’ the probability of not yet having deteriorated among those who eventually will deteriorate within 24 hours after admission to the medical emergency department. A patient could have more than one of the five individual vital signs deteriorating at the same time, therefore the total number of deteriorating vital signs (at time 0) exceed the number of deteriorating patients. Glasgow Coma Scale is not presented due to the small number of deteriorations (N = 2).

The six individual vital signs of interest contributed with 533 deteriorations among the 446 patients experiencing clinical deterioration. The most common vital sign to deteriorate was the pulse rate (194/533 deteriorations, 36.4%) followed by oxygen saturation (187/533 deteriorations, 35.1%) (Figure 3).

Oxygen saturation expressed the shortest median time to registered deterioration of 7.5 hours, (2.4–19.0 hours) whereas temperature expressed the longest median time of 12.5 hours (3.3–21.1 hours). For a detailed overview of the time of each individual vital sign deterioration, see Figure 3.

## Discussion

Although several studies have focused on risk of clinical deterioration among acutely admitted patients[5], [7], [16]–[18], studies focusing on apparently low risk patients with normal vital signs on arrival are few [3]. We show that 31% of all patients admitted to a medical emergency department with normal vital signs on arrival deteriorated within the first 24 hours and that deteriorating patients had four times higher 30-day all-cause mortality.

Previous studies have used varying definitions of clinical deterioration. As a result, most of these studies show a lower proportion of deterioration (1.7%–16.0%) than ours[5], [7], [16]–[19] and only a single study found a higher proportion of deterioration (56.5%) [20]. Some have used admission to an ICU as endpoint [7], [17], [21], others a relative comparison of risk score in each patient [5], activation of a rapid response team [22], [23] or a post hoc definition of abnormal vital signs based on the subsequent mortality [20]. These inconsistencies in outcome make it difficult to compare the results directly, and illustrate the fact that deterioration is a continuous process. Only recently, studies have begun to emphasise the need for a consensus based framework to define clinical deterioration similar to those used in ICUs [24].

We have chosen to use a less restrictive and more sensitive definition of deterioration based on the normal ranges of the individual vital signs likely to identify clinical deterioration earlier than the aforementioned strategies. We did this to investigate if these early changes in vital signs could be used to identify an at-risk patient population and/or if the nursing staff and physicians should pay extra attention to these “well appearing” patients. If we had chosen admission to the ICU within the first 24 hours after arrival to the hospital as a surrogate marker of clinical deterioration, only two patients would have been classified as having deteriorated clinically and four patients if we had chosen a cut-off of 48 hours as Kennedy et al did [17].

There is little consensus on how frequently patients should be re-evaluated after initial triage[4]–[6], [9], [10]. We found that the deteriorating vital values were registered 4 to 13 hours after arrival. The time to deterioration curves for the vital signs showed almost linear trends the first 24 hours after arrival. However, this observation is limited by the fact that the registration of vital signs was not standardised, but to a great extent were recorded at individual time frames. Few studies have previously investigated the time to clinical deterioration from arrival to the hospital. Kennedy et al. found median time to ICU transfer to be 20 hours in patients presenting at the emergency department with an infection [17] and Bleyer et al. found that approximately 25% of all deteriorations in hospitalised patients occurred within 24 hours after admission [20]. The National Institute for Health and Clinical Excellence recommends that physiological observations should be monitored at least every 12 hours, depending on decisions made at a senior level [25].

We found that 7.9% of the patients with registered clinical deterioration died within 30 days after admission. This is a low mortality rate compared to other studies [5], [20]. We believe that this is due to different definitions of deterioration, as an example Kennedy et al used transfer to the ICU as a surrogate marker, a threshold potentially more severe than single vital sign deterioration, and thereby indicating more severely ill patients [17]. Another possible explanation could be differences in case-mix between studies.

Increased 30-day mortality in deteriorating patients is probably (to some degree) preventable if the deterioration is acted upon in time, but one has to keep in mind that some fatalities are inevitable. Hogan et al. showed that poor clinical monitoring was considered the most dominant cause of preventable deaths in acute hospitals [26] and Hands observed moderate to poor adherence to monitoring procedures in patients with normal as well as abnormal vital signs [4]. This highlights the need for improved observation and follow-up. These procedures should be able to identify clinical deterioration without compromising the effectiveness of a busy emergency department.

Although a single vital sign deterioration might appear as unspecific we found large differences in the frequency of which vital sign initially deteriorated and the following 30-day mortality. Deterioration in oxygen saturation was the most frequent cause of deterioration and associated with the highest 30-day mortality. This could be because low oxygen saturation might identify a more fragile patient group, namely patients suffering from chronic respiratory diseases.

In the present study, the most frequent discharge diagnosis group among patients with normal vital signs admitted to the medical ED was “General signs and symptoms and abnormal clinical and lab findings (ICD-10: R00–R94)”. This is in concordance with a study by Schmidt et al. study regarding patients admitted to the emergency department in a similar setting as the present one [27]. We showed that the largest proportion of patients deteriorating was patients’ with a discharge diagnosis of the Respiratory system (ICD10: J00–J99). This might be because the most common individual vital sign deteriorating was oxygen saturation, and physicians assigning discharge diagnoses to the patients course of admission.

We emphasise that the present study was not an attempt to develop a clinical tool to predict and prevent morbidity and mortality in initially “well appearing” patients, but an observational study where the primary aim was to describe this patient population in the ED setting.

### Strengths and Limitations of Study

The main strength of our study is the large consecutive cohort of acute medical patients with complete follow-up for 30-day all-cause mortality. The main limitation is the absence of standardised time intervals for re-evaluation of the patients. This introduces a bias as the nurses might register vital signs more frequently in some patients (e.g. presenting at increased risk). The current work was a single-centre study from a medical ED at a university hospital, which potentially could affect the generalisability of the study. However, the hospital is the only hospital in the area and is a primary hospital for all residents in the hospital catchment area, in parallel to highly specialised tertiary functions.

The study was conducted at a medical ED describing acutely hospitalised medical patients so the risk factors’ strength of association might be different among patients hospitalised to an abdominal surgery department, cardiology patients or patients in active chemotherapy.

There is always the possibility of residual confounding, which could lead to different estimates when interpreting the independent risk factors in the multivariate models.

Due to the small number of patients died within 30 days after admission we were not able to perform subgroup analysis of mortality in the individual vital signs as well as in the different discharge categories.

## Conclusion

Among acutely admitted medical patients who arrive with normal vital signs, 31.0% deteriorated within 24 hours. Risk factors included old age (85+ years), Do-not-attempt-to-resuscitate order and if a patient was admitted from the open general ED. Thirty-day mortality among patients with deterioration was four times higher than among patients without deterioration. This pinpoints the need for an intensified follow up strategy on medical emergency patients.
